# Epigenetic age acceleration in the brain and blood of people with epilepsy and in a mouse model of focal cortical dysplasia: a retrospective cross-sectional study

**DOI:** 10.1016/j.ebiom.2026.106550

**Published:** 2026-11-09

**Authors:** Paraskevi Chasani, Mitali Katoch, Johannes R. Lemke, Rami Abou Jamra, Julia Hentschel, Till Hartlieb, Manfred Kudernatsch, Christian G. Bien, Thilo Kalbhenn, Hajo Hamer, Daniel Delev, Karl Rössler, Rafael Toledano, Antonio Gil-Nagel, Petra Mrozkova, Adam Bogdanovic, Nikola Vrskova, Jan Kudlacek, Anna Svobodova, Helena Pivonkova, Premysl Jiruska, Sarah Weckhuysen, Charissa Millevert, Viola Balaj, Manuel A. Schmid, Lucas Hoffmann, Roland Coras, Ingmar Blümcke, Katja Kobow

**Affiliations:** a Department of Neuropathology, Universitätsklinikum Erlangen, Friedrich-Alexander-Universität Erlangen-Nürnberg (FAU), Erlangen, Germany; b Institute of Human Genetics, University of Leipzig Medical Center, Leipzig, Germany; c Center for Pediatric Neurology, Neurorehabilitation and Epileptology, Schön Klinik Vogtareuth, Vogtareuth, Germany; d Research Institute “Rehabilitation, Transition, Palliation”, PMU Salzburg, Salzburg, Austria; e Department of Epileptology, Krankenhaus Mara, Bethel Epilepsy Center, Medical School OWL, Bielefeld University, Bielefeld, Germany; f Department of Neurosurgery, Medical University Vienna, Partner of the ERN EpiCARE, Vienna, Austria; g Department of Neurology, Epilepsy Center, Hospital Ruber Internacional, Madrid, Spain; h Department of Physiology, Second Faculty of Medicine, Charles University, Prague, Czech Republic; i Department of Circuit Theory, Faculty of Electrical Engineering, Czech Technical University in Prague, Prague, Czech Republic; j VIB-Center for Molecular Neurology, University of Antwerp, Belgium; k Erlangen Epilepsy Center, Universitätsklinikum Erlangen, Friedrich-Alexander-Universität Erlangen-Nürnberg (FAU), Erlangen, Germany; l Department of Neurosurgery, Universitätsklinikum Erlangen, Friedrich-Alexander-Universität Erlangen-Nürnberg (FAU), Partner of the ERN EpiCARE, Erlangen, Germany; m Center for Rare Diseases, University of Leipzig Medical Center, Leipzig, Germany; n Center for Neurosurgery and Epilepsy Surgery, Schön Klinik Vogtareuth, Vogtareuth, Germany; o Department of Neurosurgery - Epilepsy Surgery (Evangelisches Klinikum Bethel), Medical School OWL, Bielefeld University, Bielefeld, Germany; p Department of Neurology, Antwerp University Hospital, Antwerp, Belgium; q Translational Neurosciences, Faculty of Medicine and Health Science, University of Antwerp, Antwerp, Belgium; r Epilepsy Research Centre Prague, Czech Republic

**Keywords:** Ageing, Structural brain lesion, Genetic epilepsy, Epigenetic clock, DNA methylation

## Abstract

**Background:**

People with epilepsy have reduced life expectancy and more age-related comorbidities, suggesting a link to faster biological ageing. Direct molecular evidence has been scarce.

**Methods:**

We applied DNA-methylation-based epigenetic clocks to brain and blood samples from 324 unique individuals with structural or genetic epilepsy and 65 non-epilepsy controls, and to an *in utero* electroporation mouse model of somatic mTOR pathway activation (etiocopy of human FCD type 2) sampled before and after seizure onset. Epigenetic age acceleration was defined as the residual from a robust regression on chronological age, sex, cell-type composition, and array type. Relative telomere length was measured in the mouse model to independently validate biological ageing.

**Findings:**

In brain, epigenetic age acceleration was consistent across structural lesion types (median +7.3 years, *p* < 0.0001), most pronounced in GG (+10 years, *adj. p* < 0.0001), followed by MOGHE (+8.1 years, *adj. p* < 0.0001), TLE (+6.6 years, *adj. p* < 0.001), and FCD (+5.6 years, *adj. p* < 0.001). In blood, age acceleration was not significant after adjustment for cell composition, age, and sex (median +0.7 years, *p* = 0.20), except in FCD (+2.3 years, *adj. p* < 0.05). Across structural epilepsies, acceleration was greater in brain than in blood (median +7.3 vs. + 1.4 years, *p* < 0.0001). Available VPA-exposure data (n = 175) was not significantly associated with age acceleration. In a drug-naïve mTOR mouse model, mutant epileptic brains, but not blood, showed epigenetic age acceleration (*adj. p* < 0.05). Ageing signatures were already detectable in brains of animals in the pre-seizure latency phase and were independently supported by shortened relative telomere length (*adj. p* = 0.05), indicating that biological ageing can precede overt epilepsy.

**Interpretation:**

Accelerated epigenetic ageing is a consistent feature of the epileptic brain in drug-resistant structural epilepsies and a candidate pathway for disease modification. The contribution of systemic ageing remains unresolved, and generalisability to other aetiologies will need to be further tested.

**Funding:**

Else Kröner Fresenius Stiftung, German Science Foundation, L’Institut pour la Recherche en Santé Publique, Interdisziplinäres Zentrum für Klinische Forschung, Czech Science Foundation, Ministry of Health of the Czech Republic, Ministry of Education, Youth and Sports of the Czech Republic, Research Foundation–Flanders FWO.

**Research in context**

**Evidence before this study**

Prior work, including our own, showed altered DNA methylation and other epigenetic changes in human temporal lobe epilepsy and other epilepsies. Molecular, cellular, imaging, cognitive, and histological studies have suggested premature ageing in the epileptic brain, but not using DNA methylation-based measures. Epidemiological studies further showed robust associations between epilepsy, age-related disease and multimorbidity, and excess mortality in the general population. Quantitative estimates of biological age acceleration using DNA methylation-based epigenetic clocks in human drug-resistant surgical brain tissue across distinct structural aetiologies, within-individual brain and blood comparisons, and integration with a mechanistic drug-naïve animal model sampled before and after seizure onset, have not been reported.

**Added value of this study**

We show that epigenetic age acceleration is a consistent and pronounced feature of epileptic brain tissue in drug-resistant structural epilepsies, spanning several human lesion types and an mTOR-driven mouse model, whereas genetic epilepsies examined in blood do not show comparable acceleration at the group level. Cross-tissue analyses reveal that ageing effects are generally stronger in brain than in blood, with focal cortical dysplasia as the only subgroup showing similar age acceleration in both tissues, pointing to possible systemic ageing in this aetiology. By sampling an mTOR-driven model before and after seizure onset, we identify epigenetic ageing signatures and epilepsy-linked methylation modules that are to some extent already present in some animals during the latency phase, suggesting that accelerated epigenetic ageing can precede overt epilepsy. Together, these data extend previous evidence of epigenetic dysregulation in epilepsy by providing quantitative, epilepsy subtype- and tissue-resolved measures of biological age acceleration, and are consistent with clinical observations of premature mortality and multimorbidity.

**Implications of all the available evidence**

Taken together with existing literature on comorbidities, premature ageing-related molecular and cellular changes, and epigenetic dysregulation in epilepsy, our findings support the view that structural epilepsies are associated with accelerated biological ageing of the brain, with more heterogeneous and aetiology-dependent changes in peripheral blood. This work suggests that ageing-related pathways captured by epigenetic clocks could contribute to epileptogenesis and to the excess burden of age-related disease in people with epilepsy, while also highlighting substantial heterogeneity across aetiologies and tissues. In the near term, epigenetic clocks and methylation networks may serve as integrative biomarkers to stratify risk and to monitor candidate disease-modifying interventions targeting ageing pathways. In the longer term, rigorous longitudinal and interventional studies will be needed to determine whether modulating biological ageing can meaningfully alter epilepsy onset, progression, and the development of age-related comorbidities.

## Introduction

Biological age (defined by molecular and cellular states), as opposed to chronological age (defined by time from birth), can be estimated using epigenetic clocks based on DNA methylation patterns, which change predictably with age and are influenced by genetic, environmental, and lifestyle factors. The most widely recognised epigenetic clock, developed by Horvath et al.,^1^ has significantly changed the current understanding of the ageing process at a molecular level. Accelerated biological ageing has been associated with several age-related diseases, including cardiovascular diseases,^2^ Alzheimer's disease,^3^ psychiatric disorders,^4^ and cancer,^5^ and outperforms chronological age in predicting disease risk.^6^

Life expectancy in people with epilepsy is reduced by more than a decade compared to the general population.^7^ However, excess mortality can only in part be attributed to Sudden Unexpected Death in Epilepsy (SUDEP), accidents (e.g., falls), or suicide. Epidemiological studies show that adults with epilepsy have higher rates of dementia, cerebrovascular and cardiovascular disease, diabetes, chronic lung disease, arthritis, and osteoporosis than the general population, even after accounting for some shared risk factors. This clustering of age-related comorbidities raises the possibility that epilepsy is linked to altered biological ageing, either through brain-specific or systemic mechanisms.^8^ Consistent with this hypothesis, seizures have been linked to molecular and cellular changes characteristic of ageing, including chromosomal instabilities,^9^, ^10^ telomere attrition,^11^ epigenomic changes,^12^, ^13^ oxidative stress,^14^ and cellular senescence,^15^ suggesting a seizure-driven mechanism of premature brain ageing across diverse epilepsy syndromes, although direct evidence from human tissues has been limited. Clinically, people with epilepsy exhibit an increased burden of age-related comorbidities,^16^ accelerated cognitive decline,^17^ changes in brain volume and cortical thinning^18^, ^19^, ^20^ (reference^19^ is a preprint), and histopathological signs of faster brain ageing.^21^ Yet, while epigenetic dysregulation has been described in human^10^, ^13^, ^22^, ^23^, ^24^ and experimental epilepsy,^25^, ^26^, ^27^ biological age acceleration in the epileptic brain and blood has not been quantified using DNA methylation-based epigenetic clocks. Epilepsy is one of the most common severe and chronic neurological disorders affecting people of all age groups.^28^ It is characterised by spontaneous recurrent seizures, whereby the onset and progression of seizure activity remain difficult to predict in affected patients, irrespective of their epileptogenic condition (e.g., structural, genetic, infectious, metabolic, or immune).^29^ About 1% of the world's population suffers from epilepsy. Approximately 30% of patients lack response to currently available antiseizure medications, highlighting the unmet clinical need for novel treatment strategies.^30^ Notably, interventions that slow biological ageing, such as caloric restriction, exercise, and certain drugs, not only mitigate age-related disease risk but also show efficacy in epilepsy.^31^, ^32^, ^33^, ^34^, ^35^

Here, we applied species-specific pan-tissue epigenetic clocks to brain and blood samples from patients with structural epilepsies and genetically driven developmental and epileptic encephalopathies, as well as to an *in utero* electroporation mouse model of mTOR-driven epilepsy (Fig. 1). We asked whether epigenetic age is accelerated in epilepsy, varies by tissue and aetiology, and precedes spontaneous seizure onset. We hypothesised that age acceleration would differ between epilepsy and control groups, vary across tissues and aetiologies, and be detectable in the mouse brain before and after seizure onset. Our findings extend previous evidence of epigenetic dysregulation in epilepsy by providing quantitative, tissue- and aetiology-resolved measures of biological age acceleration and identify biological ageing as a potentially relevant pathway in epilepsy that warrants investigation as a target for disease modification.

**Fig. 1:**
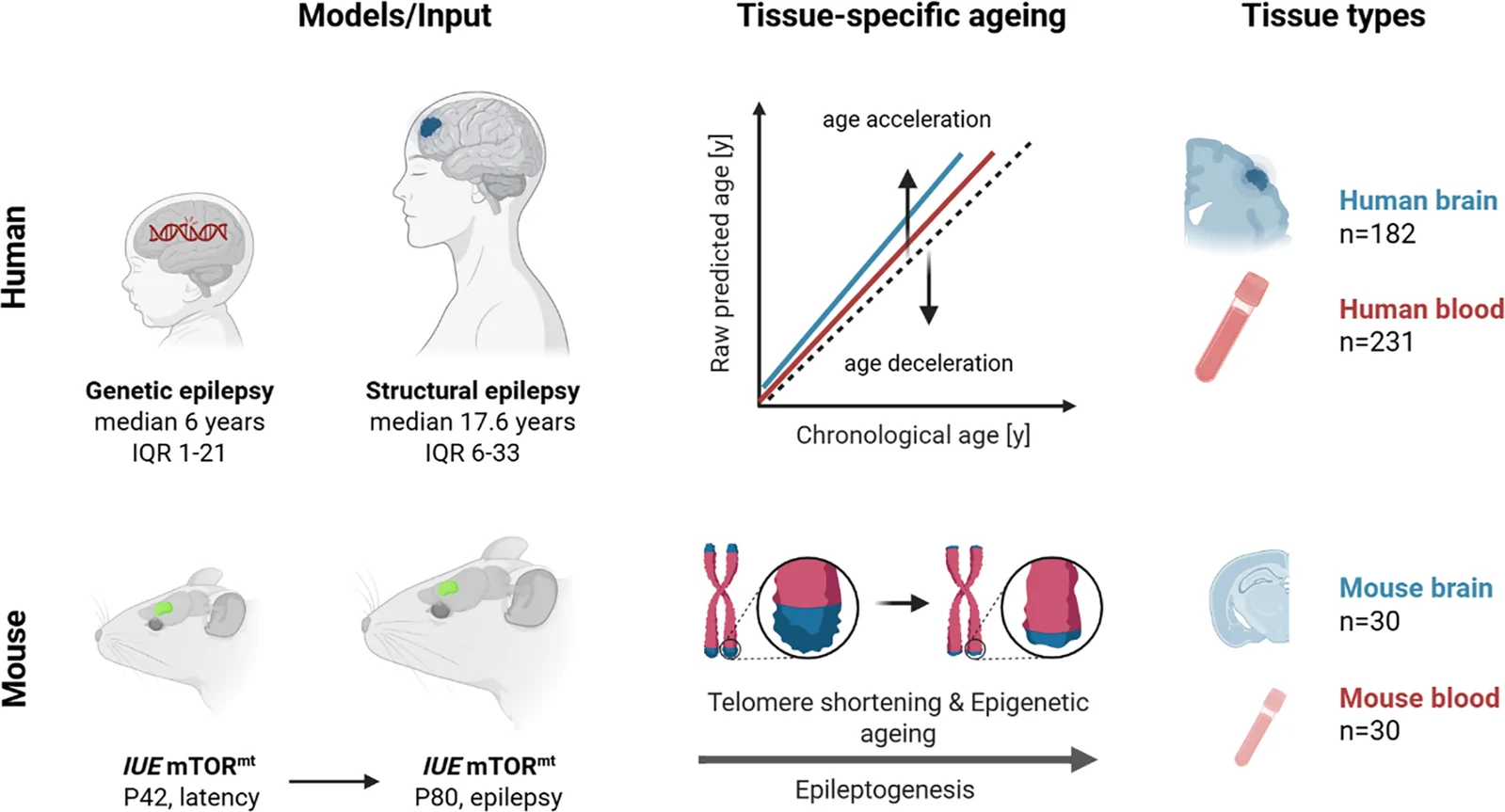
**Graphical abstract of the study design.** Tissue-specific ageing was investigated in human structural and genetic epilepsies and a mouse model of mTOR-associated epilepsy using DNA methylation-based age estimates and telomere length measurements. Blue and red denote brain and blood, respectively. IQR, interquartile range. IUE, *in utero* electroporation. mTOR, mechanistic target of rapamycin. mt, mutant. P, postnatal day.

## Methods

### Human sample cohort

This retrospective cross-sectional study is reported in accordance with the STROBE guidelines (see completed STROBE checklist).

### Surgical brain samples

We included 162 individuals who underwent surgery for the treatment of their focal drug-resistant epilepsy and were diagnosed with focal cortical dysplasia type 2A/B (FCD, *n* = 52), mild malformation of cortical development with oligodendroglial hyperplasia in epilepsy (MOGHE, *n* = 48), temporal lobe epilepsy with hippocampal sclerosis (TLE, *n* = 32), or ganglioglioma (GG, *n* = 30) based on clinical presentation, MRI, and histology. No randomisation was possible due to the study's retrospective design, and sample size was limited by high-quality tissue availability in the participating centres and by our inclusion criteria (centralised tissue work-up and histology) rather than by a priori power calculation. Routine diagnostic tissue workup, histopathology, and clinical phenotypes have been described previously.^36^, ^37^, ^38^, ^39^, ^40^

All structural epilepsy cases underwent standard neuropathological assessment, including Haematoxylin-Eosin staining and cell-type and antigen-specific immunohistochemistry on 4 μm-formalin-fixed paraffin-embedded (FFPE) sections. Expert neuropathologists (RC, IB) confirmed diagnoses using the International League Against Epilepsy (ILAE) classification systems.^40^, ^41^ DNA was extracted from microdissected lesional areas and processed for DNA methylation profiling with Illumina Infinium Methylation EPICv1 or EPICv2 BeadChips (Illumina, Germany). We additionally included 20 autopsy brain controls without epilepsy or other neurological disease, all with non-neurological causes of death (respiratory, cardiovascular, oncological) and no cortical architectural abnormalities. All samples were in-house controls, matched for preparation from frontal and temporal lobes, and underwent the identical centralised workup, including sample preparation, DNA extraction, and array hybridisation. Cohort characteristics are summarised in Table 1.

**Table 1:** Clinical data summary.

	Aetiology	Dataset	#	VPA Y/N	F/M	Age (IQR)	Onset (IQR)	Duration (IQR)	Array
Human brain									
CTRL	autopsy	GSE185090, GSE156374, GSE227239	20	NA	12/8	57 (55–71)	NA	NA	Human EPICv1+2
FCD	structural		52	31/12	21/31	19 (6–32)^a^	4 (0.5–5)	11 (4–25)	
MOGHE	structural		48	38/8	20/28	7 (4–18)^a^	1 (0.3–3)	5 (3–15)	
TLE	structural		32	16/11	15/17	39 (26–42)^a^	6 (1–16)	21 (10–30)	
GG	structural		30	3/22	9/21	18.5 (11–28)^a^	12 (4–15)	3 (1–13)	Human EPICv2
Human blood									
CTRL	biopsy	GSE182991, GSE191276, E-MTAB-12728	45	NA	22/23	34 (8–44)	NA	NA	Human EPICv1+2
FCD	structural		27	2/4	15/12	24 (19–33)	4 (2.5–7)	16 (9–25)	
MOGHE	structural		6		3/3	13 (6–22)	4 (2–7)	8 (5–12)	
TLE	structural		24		15/9	41 (34–49)^a^	14 (6–22)	27 (15–32)	
SCN1A	genetic		56	14/-	31/25	7 (2–22)^a^	0 (0–0)	3 (0–13)	Human EPICv2
SCN2A	genetic		11		3/8	5 (1–12)^a^	1 (0–3)	4 (0–15)	
SCN8A	genetic		18		11/7	3 (0–7)^a^	0 (0–0)	0 (0–1)	
KCNQ2	genetic		27	5/5	13/14	6 (0–16)^a^	0 (0–0)	3 (0–12)	
STXBP1	genetic		17	4/-	6/11	19 (1–24)^a^	0 (0–0)	10 (1–23)	

CTRL, control; F, female; FCD, focal cortical dysplasia; GG, ganglioglioma; IQR, interquartile range; KCNQ2, Potassium Voltage-Gated Channel Subfamily Q Member 2; M, male; MOGHE, mild malformation of cortical development with oligodendroglial hyperplasia in epilepsy; N, no; NA, not applicable; SCN1/2/8A, Sodium Voltage-Gated Channel Alpha Subunit 1/2/8; STXBP1, Syntaxin-Binding Protein 1; TLE, temporal lobe epilepsy; VPA, valproic acid; Y, yes; #, number.

a Adjusted *p*-value < 0.05 (significantly different chronological age from respective controls).

### Blood samples

Blood samples were obtained from 129 patients with developmental and epileptic encephalopathy (DEE) caused by pathogenic or likely pathogenic variants in Sodium Voltage-Gated Channel Alpha Subunit 1, 2 or 8 (*SCN1A*, n = 56; *SCN2A*, n = 11; *SCN8A*, n = 18), Syntaxin-Binding Protein 1 (*STXBP1*, n = 17), and Potassium Voltage-Gated Channel Subfamily Q Member 2 (*KCNQ2*, *n* = 27), and from 57 patients with structural focal epilepsy (FCD, n = 27; MOGHE, n = 6; TLE, *n* = 24). Healthy controls (n = 45) comprised 15 samples from previously published datasets (GSE182991, GSE191276, E-MTAB-12728) and 30 unaffected parents. Genomic DNA was extracted from whole blood using the EZ1&2 DNA Blood Kit and EZ1 extraction robot (Qiagen, Germany) per manufacturer's instructions. Bisulfite conversion and hybridisation to Illumina Infinium Human Methylation EPIC v1 or v2 arrays were performed at the Life & Brain Research Centre, Bonn. Clinical data are summarised in Table 1.

### Mouse model

Wild-type C57BL/6 mice (Charles River, both sexes, n = 30) were housed in groups under standard conditions (22 ± 1 °C, 12 h light/dark cycle). Animals were randomly assigned into five groups, i.e., P42-sham, P42-mTORᵐᵗ (latency), P80-sham, P80-mTORᵐᵗ without seizures (prolonged latency), and P80-mTORᵐᵗ with seizures (epilepsy; Table 2). Methodological details on FCD induction, electrode implantation, and tissue preparation are provided in the Supplements.

**Table 2:** Animal data summary.

	Aetiology	#	F/M	Epilepsy (Y/N)	Array
Brain & blood					
P42-sham	structural	5	2/3	N	Mouse EPICv1
P42-mTOR^mt^		7	2/5	N	
P80-sham		6	0/6	N	
P80-mTOR^mt^		6	4/2	N	
P80-mTOR^mt^ with seizures		6	3/3	Y median sz onset (IQR) P50.5 (P47-58)	

F, female; IQR, interquartile range; M, male; mTOR, mammalian target of rapamycin; #, number; N, no; P, postnatal day; sz, seizure; Y, yes.

### Age prediction with epigenetic clocks

Genome-wide DNA methylation was profiled in human and mouse brain and blood using species-specific Illumina BeadChip platforms (Human Infinium Methylation EPIC v1/v2; Mouse Infinium Methylation BeadChip). For EPICv2 probes interrogating the same CpG site, the probe appearing first in the Illumina manifest was used. Raw signal intensity data (.idat files) were imported into R (v4.5.1) using the ‘minfi’ (v1.54.1) or ‘SeSAMe’ package (v1.26.0; for mouse data). For both human and mouse datasets, quality control included assessment of median signal intensities, detection *p*-values, and predicted sex to identify and exclude low-quality samples.

Background correction and dye-bias normalisation were performed using the noob (normal-exponential out-of-band) method, which also incorporates detection p-value masking. For human data, we removed probes with detection *p*-values > 0.01, very low signal intensity, sex chromosome probes, non-CpG or control probes, unreliable probes from published annotations, or those overlapping common SNPs. Mouse data underwent additional species- and strain-specific masking to account for genetic variation within the C57BL/6 strain, exclusion of poorly designed and multimapping probes, and inference of Infinium-I probe channel designations for dye-bias correction. Normalised *β*-values were calculated using the getBetas() function. To account for cellular heterogeneity, reference-based cell-type deconvolution was applied. Neuronal and glial cell-type proportions were estimated using the ‘HiBED’ package (v0.99.15).^42^ Leucocyte subtype proportions were inferred using the ‘EpiDISH’ package (v2.24.0) with the centDHSbloodDMC.m reference matrix and the robust partial correlations (RPC) method.

In human brain and blood samples, biological age was estimated with the principal component Horvath pan-tissue clock (PCHorvath1) using the ‘PCclock’ package (v0.1.0). Rather than using individual CpG probes directly, PCHorvath1 projects each sample’s methylation profile onto a principal component space derived from the original Horvath clock training data and uses the top 30–50 components to predict age, improving robustness to technical variation and platform differences (e.g., EPIC v1 vs. v2) and making the clock well suited to multi-cohort archival data. CpGs that were entirely missing across all samples were imputed using a predefined reference mean, whereas sporadically missing values were replaced by the mean methylation level of the respective CpG across the available samples in the dataset. Epigenetic age acceleration was defined per tissue as the residual from robust linear regression in control samples using the lmRob() function from the ‘robustbase’ package (v0.99.4.1), regressing predicted age on chronological age, sex, estimated cell-type proportions, and array type as covariates, and then applying the fitted model to all samples of the corresponding tissue. Age acceleration was taken as the residual (observed minus expected predicted age). Positive values indicate acceleration, and negative values indicate deceleration.

In mouse samples, epigenetic age (in months) was predicted using the ‘SeSAMe’ predictAge() function based on a murine clock built from 347 MM285 probes.^43^ Again, epigenetic age acceleration was derived as residuals from a robust linear regression of predicted age on chronological age, fitted in sham-treated controls and then applied to all mouse samples. CpGs with missing methylation values were excluded on a per-sample basis, and age estimates were calculated using the remaining available clock CpGs. Adjusted age estimates were converted to days for visualisation. One blood outlier, identified by within-tissue z-scores, was excluded.

### Consensus network analysis with WGCNA

To identify modules of co-methylated CpG sites associated with biological traits, we performed weighted gene correlation network analysis (WGCNA) using the ‘*WGCNA*’ package (v1.73). Sex-chromosome and non-CpG probes were excluded, followed by removal of probes with frequent missingness (≥1% of samples). Residual missing values (<0.01%) were imputed using the mean *β*-value of the corresponding probe across the available samples before M-value transformation. We then retained the top 25% most variable probes, and transformed *β*-values to M-values to stabilise variance prior to network construction. An unsigned weighted network was constructed using pairwise biweight midcorrelation, with soft-thresholding chosen using the pickSoftThreshold() function to approximate scale-free topology (R^2^ > 0.85). Modules were defined by hierarchical clustering of the topological overlap matrix (TOM) and dynamic tree cutting (cutreeDynamic with a minimum module size of 100), with highly correlated modules merged (eigengene correlation > 0.4). Modules were labelled by colour and summarised by their first principal component (module eigengene). Associations between module eigengenes and sample traits (epilepsy, latency, sham, age) were assessed using Pearson correlation. Modules significantly associated with epilepsy or age (*adj. p* < 0.05) were selected for downstream analysis. Modules were retained for downstream analysis only if they showed a significant and trait-specific association with epilepsy or age after FDR correction (*adj. p* < 0.05). Modules positively correlated with epilepsy and negatively correlated with the sham/control, or vice versa, were prioritised, as this opposing pattern provides stronger evidence of disease specificity than association with a single trait alone.

Module CpGs were mapped to genes (mm10 mouse genome) using strand-aware promoter-overlap (±1 kb around transcription start sites) and nearest-gene annotation to ensure comprehensive non-redundant gene-level representation of each module. Reactome pathway enrichment analysis was performed using the ‘ReactomePA’ package (v1.52.0) with a hypergeometric test and multiple testing correction with Benjamini-Hochberg, and significantly enriched pathways (*adj. p* < 0.05) were used to interpret module function. To visualise functional relationships within modules, pathway-level networks were constructed in Cytoscape (v3.10.3), with nodes representing enriched Reactome pathways and edges reflecting gene overlap between pathways. Node size was scaled according to the number of genes contributing to each pathway, and edge width was proportional to the degree of gene overlap between pathways. Circos plots summarising pathway overlap among epilepsy- or age-related modules (significance cut-offs at *adj. p* < 0.01 and *adj. p* < 0.05) were also generated using the ‘circlize’ package (v0.4.16). Heatmaps of relative methylation (row-scaled *β*-values) were generated for CpG probes in key modules using the ‘ComplexHeatmap’ package (v2.24.1), grouped by trait categories to highlight methylation shifts associated with epilepsy or age. The graphical abstract was created with BioRender.

### Ethics

All human blood and tissue samples were obtained under written informed consent from patients or their legal guardians. Study protocols adhered to the principles of the 2024 Declaration of Helsinki and were approved by the Ethics Committee of the Medical Faculty of the Friedrich-Alexander-Universität Erlangen-Nürnberg, Germany (92_14B, 193_18B, and 18–193_1-Bio), the University of Leipzig (224/16-ek, 402/16-ek), the University of Münster (2015-088-f-S), and the University of Antwerp (18/38/415).

All animal experiments complied with the Animal Protection Law of the Czech Republic and EU Directive 2010/63/EU, were conducted in accordance with the ARRIVE 2.0 guidelines (see completed ARRIVE checklist) and were approved by the Ethics Committees of the Second Faculty of Medicine and of the Ministry of Education, Youth, and Sports (Project Licence No. MSMT-31765/2019-4 and MSMT-20122/2023-6).

### Statistics

All statistical analyses were performed in R (v4.5.1). Normality was assessed using the Shapiro–Wilk test and visual inspection of histograms, and equality of variances was tested with Levene's test. Robust linear regression models were fitted to estimate the relationship between predicted epigenetic age and chronological age, with models trained on control samples. Cell-type proportions, chronological age, sex, and array version were included as covariates in human analyses, whereas age alone was used in mouse analyses. Residuals were used as the measure of epigenetic age acceleration.

Group differences in age and age acceleration were tested using non-parametric tests (Mann–Whitney U for two groups, Kruskal–Wallis with Dunn’s post hoc test and Benjamini-Hochberg correction for multiple groups). Within-patient brain–blood differences were tested with paired t-tests. Associations with clinical variables (age at epilepsy onset, disease duration, seizure burden) were assessed by Spearman correlation. The effect of prior valproate (VPA) exposure on epigenetic age acceleration was tested by Mann–Whitney U test on brain and blood residuals from individuals with documented VPA-exposure status, without and with additional adjustment for diagnosis. Module-trait correlations in WGCNA were assessed by Pearson correlation with FDR adjustment. All tests were two-sided, and adjusted p < 0.05 was considered significant.

No a priori power calculation was performed for the mouse experiments. Adequacy of group sizes was assessed with the resource equation method,^44^ giving *E* = 25 for the five allocation groups and *E* = 27 for the three analysis groups, both above the recommended range of 10–20. A sensitivity analysis (80% power, two-sided *α* = 0.05) gave minimum detectable standardised effects of *d* = 1.52 for epileptic vs. sham animals and *d* = 1.20 for latency vs. sham animals. The mouse arm was therefore powered to detect large effects only.

### Role of funders

Funders had no role in study design, data collection, analysis, interpretation, or writing of the manuscript.

## Results

### Accelerated biological ageing in the human epileptic brain

To test whether epilepsy is associated with accelerated ageing, we applied epigenetic clocks to resected brain tissue from 162 patients with drug-resistant epilepsy due to structural brain lesions (median chronological age 18 years, IQR 6–33) and 20 nonepileptic autopsy controls without a history of neurological disorders or seizures (median age 57 years, IQR 55–71, Mann–Whitney U test, *p* < 0.0001; Table 1).

The epilepsy cohort included major lesion classes associated with drug-resistant focal epilepsy: focal cortical dysplasia (FCD, *n* = 52, median age 18.5 years, IQR 5.8–32, Dunn test with Benjamini-Hochberg correction, *adj. p* < 0.0001), mild malformation of cortical development with oligodendroglial hyperplasia in epilepsy (MOGHE, *n* = 48, median age 6.8 years, IQR 4–18.2, *adj. p* < 0.0001), temporal lobe epilepsy with hippocampal sclerosis (TLE, *n* = 32, median age 39 years, IQR 25.8–42, *adj. p* < 0.01), and ganglioglioma (GG, *n* = 30, median age 18.5 years, IQR 10.5–28.3, *adj. p* < 0.0001; Table 1, Supplementary Fig. S1A). All patients underwent brain surgery for seizure treatment. Both sexes were included. Because lesion types were associated with distinct age distributions and suitable control tissue was limited by availability and quality, the age distributions of the epilepsy subgroups could not be matched to those of the nonepileptic autopsy controls.

Benchmarking four different pan-tissue or blood-specific clocks, PCHorvath1, a principal component-based implementation of Horvath’s pan-tissue clock designed to improve robustness across array types and datasets, provided the most accurate age estimates across tissue types in controls (robust linear regression: R^2^ = 0.9, robust residual standard error = 4.2 years, root mean square error of raw predictions = 5.21 years; Supplementary Fig. S1B–D, Fig. 2A). Age acceleration in epilepsy was defined as the residual from robust linear regression of predicted age on estimates of neuron-glia composition, chronological age, sex, and array type as covariates, with the resulting standardised model residuals defined as age acceleration. However, direct comparisons of raw predicted and chronological age plots per diagnosis are also shown (Fig. 2B). Across all structural epilepsy samples, median age acceleration relative to non-epilepsy controls was 7.3 years (IQR 4–11.8, Mann–Whitney U test, *p* < 0.0001). When stratified by lesion type, age acceleration was most pronounced in GG (10 years, IQR 5.1–13.6, Dunn test with Benjamini-Hochberg correction, *adj. p* < 0.0001) followed by MOGHE (8.1 years, IQR 6.5–12.6, *adj. p* < 0.0001), TLE (6.6 years, IQR 1.3–11.8, *adj. p* < 0.001), and FCD (5.6 years, IQR 1.7–10.2, *adj. p* < 0.001; Fig. 2C). In a subset of patients, age acceleration was exceptionally high, up to 40 years. Accelerated ageing was also observed in histologically normal epileptic temporal neocortex of patients with TLE (verified independently by two experienced neuropathologists, authors RC, IB), indicating that the effect is not solely attributable to overt lesion pathology.

**Fig. 2:**
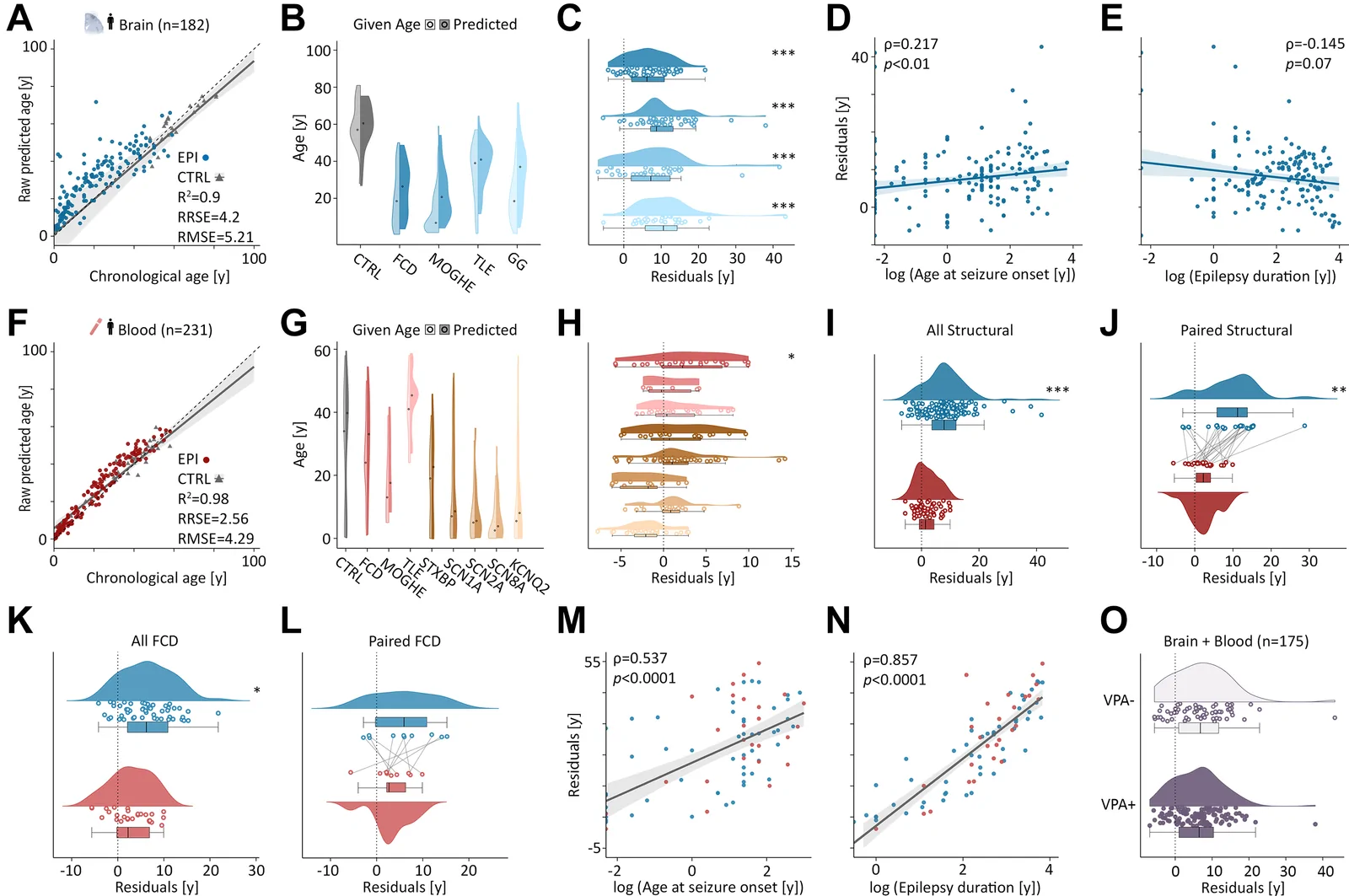
**Biological ageing in the epileptic brain and blood.** (**A**) Correlation between predicted and chronological age in surgical brain tissue samples, including patients with focal structural epilepsy (*blue*) and no-epilepsy autopsy controls (*grey*). (**B**) Raw predicted age plotted against chronological age per diagnosis, without covariate correction. (**C**) Age acceleration, defined as the residual from a robust linear regression including chronological age, sex, neuron-glia composition, and array type covariates, per diagnosis. Colour scheme in B also applies to C. (**D**) Age acceleration plotted against log-transformed age at onset and (**E**) log-transformed epilepsy duration. (**F**) Correlation of predicted and chronological age in blood samples obtained from patients with epilepsy (*red*), including structural and genetic epilepsies, and no-epilepsy controls (*grey*). (**G**) Age distributions (given vs. raw predicted age) and (**H**) age acceleration (i.e., residuals) relative to controls are shown per disease group. Colour scheme in G also applies to H. (**I**) Age acceleration comparison across tissue types, i.e., blood (*red*) and brain (*blue*) in all available structural epilepsies and (**J**) within-patient brain–blood sample pairs (connected by line) both sampled together on the day of surgery. (**K**) Age acceleration comparison across tissue types in all FCD and (**L**) within-patient FCD brain–blood sample pairs (connected by line). (**M**) Age acceleration in all available FCD samples, including brain (*blue*) and blood (*red*) plotted against log-transformed age at onset and (**N**) log-transformed epilepsy duration. (**O**) Age acceleration in patients treated at any given time point prior to surgery with valproate (VPA+) and those never treated with valproate (VPA−). Colour scheme for FCD samples in B and C, G and H also applies to K–N. CTRL, control, FCD, focal cortical dysplasia, GG, ganglioglioma, KCNQ2, Potassium Voltage-Gated Channel Subfamily Q Member 2, MOGHE, mild malformations of cortical development with oligodendroglial hyperplasia in epilepsy, SCN1A, Sodium Voltage-Gated Channel Alpha Subunit 1, SCN2A, Sodium Voltage-Gated Channel Alpha Subunit 2, SCN8A, Sodium Voltage-Gated Channel Alpha Subunit 8, STXBP1, Syntaxin-Binding Protein 1, TLE, temporal lobe epilepsy, VPA, valproic acid.

Epigenetic age acceleration in the brain significantly correlated with age at epilepsy onset (Spearman ρ = 0.22, *p* < 0.01; Fig. 2D) but not disease duration (ρ = −0.145, *p* = 0.07; Fig. 2E).

Taken together, these findings support accelerated biological ageing in the human epileptic brain.

### Accelerated biological ageing in the blood of patients with epilepsy

To assess whether age acceleration in epilepsy extends beyond the brain, we applied the PCHorvath1 clock to blood samples from 186 patients with epilepsy with structural or genetic aetiologies (median age 13.8 years, IQR 2–30) and 45 controls (median age 34 years, IQR 8–44, Mann–Whitney U test, *p* < 0.001; Table 1) using harmonised preprocessing.

Age acceleration was estimated using a generalised additive model that included estimates of blood cell composition, sex, and chronological age as covariates. Standardised model residuals from this model were defined as age acceleration. At the group level, blood-derived epigenetic age did not show consistent acceleration in epilepsy compared with controls (median 0.7 years, IQR -1.6-3.0, Mann–Whitney U test, *p* = 0.20, Fig. 2F–G). Significant age acceleration was detected only in FCD (median 2.3 years, IQR -0.2 to 6.9, Dunn test with Benjamini-Hochberg correction, *adj. p* < 0.05; Fig. 2H), but not in MOGHE, TLE, or DEEs caused by pathogenic or likely-pathogenic variants in Syntaxin-Binding Protein 1 (*STXBP1*), Sodium Voltage-Gated Channel Alpha Subunit 1 (*SCN1A*), Sodium Voltage-Gated Channel Alpha Subunit 2 (*SCN2A*), Sodium Voltage-Gated Channel Alpha Subunit 8 (*SCN8A*), and Potassium Voltage-Gated Channel Subfamily Q Member 2 (*KCNQ2*).

Cross-tissue comparisons in all structural epilepsy cases revealed significantly greater age acceleration in brain than in blood (brain, n = 162: median 7.3 years, IQR 4–11.8; blood, n = 57: median 1.4 years, IQR -0.8-4.4, Mann–Whitney U test, *p* < 0.0001; Fig. 2I). This was confirmed in a subsequent within-subject paired brain–blood comparison (n = 24; paired t-test, *p* < 0.01; Fig. 2J).

Lesion-specific analyses confirmed greater age acceleration in brain than in blood for FCD (n = 69 unique patients contributing brain, blood, or paired samples, Mann–Whitney U test with Benjamini-Hochberg correction, *adj. p* < 0.05; Fig. 2K). This difference was not significant in the within-patient paired FCD subgroup (n = 10, paired t-test, *p* = 0.43; Fig. 2L). In FCD, age acceleration was significantly correlated with age at epilepsy onset (Spearman ρ = 0.537, *p* < 0.0001; Fig. 2M) and disease duration (ρ = 0.857, *p* < 0.0001; Fig. 2N). Because disease duration is strongly collinear with chronological age and these correlations combined brain and blood samples, they should be considered exploratory and may partly reflect residual age structure rather than independent effects of epilepsy duration. Detailed clinical data on seizure types, seizure frequency, cumulative seizure burden, antiseizure medication (ASM) history, and comorbidities were not systematically available in our combined brain and blood cohort. Where documented (n = 175), VPA exposure was not significantly associated with age acceleration (Mann–Whitney U test, *p* = 0.55; Fig. 2O), and remained non-significant after adjustment for diagnosis (*p* = 0.58). Moreover, the largely VPA-naïve benign GG cohort showed the highest acceleration, arguing against VPA as the primary explanation, though causal attribution remains constrained (Fig. 2B and C).

Our data indicate that epigenetic age acceleration in epilepsy is predominantly brain-specific, with limited evidence for systemic effects in blood. FCD was the only subgroup with significant age acceleration in both tissues and no detectable difference between them.

### Accelerated biological ageing is species-independent

We next examined a mouse model of mTOR-driven FCD generated by *in utero* electroporation (*IUE*) of a pathogenic somatic variant in the mammalian target of rapamycin (mTOR) gene (Supplementary Fig. S2A and B). This etiocopy model recapitulates key features of human FCD, including its aetiology, disease course, and histopathological representation of the developmental brain lesion. We examined DNA methylation-based estimates of biological ageing in brain and blood tissues at two disease stages: postnatal day 42 (P42, representing the clinically silent latency phase) and postnatal day 80 (P80, chronic epilepsy). Age-matched sham-treated mice served as controls. All animals were implanted with cortical electrodes at day 35, followed by a 5-day recovery period and continuous video-EEG monitoring for at least 2 days (P42 cohort) or up to 38 days (P80 cohort; Supplementary Fig. S2A and C). By P80, about 50% of mTOR-mutant mice developed spontaneous recurrent seizures. A subset of P80 mTOR-mutant mice did not exhibit seizures and, for further analysis, was classified as remaining in a prolonged latency phase.

Biological age in mice was assessed using a murine ageing model based on 347 CpG sites. Brains of epileptic mice showed significant epigenetic age acceleration compared to controls (Dunn test with Benjamini-Hochberg correction, *adj. p* < 0.05; Fig. 3A), whereas matched blood samples showed no significant group differences (*adj. p* = 0.34; Fig. 3A). Latency animals did not show consistent brain age acceleration, although ageing-related methylation changes were detectable in some animals before seizure onset.

**Fig. 3:**
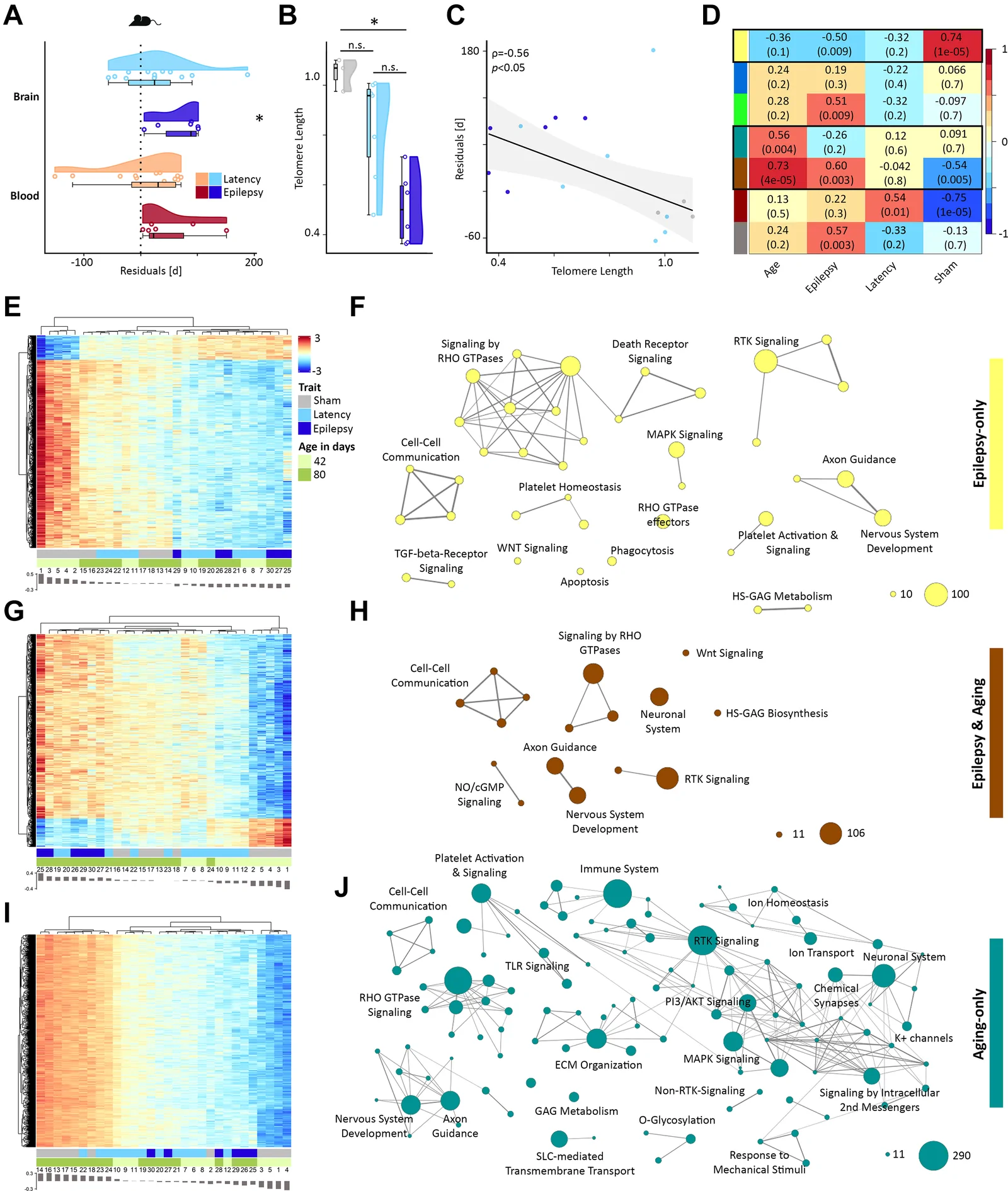
**Biological ageing in a somatic mTOR-mutant mouse model.** (**A**) Tissue- and trait-specific age acceleration of mTOR-mutant epileptic mice (*dark blue, dark red*; epilepsy) and mTOR-mutant nonepileptic mice (*light blue, light red*; latency) relative to controls (dotted line). (**B**) Relative telomere length estimates in sham controls, latency, and epileptic animals. (**C**) Correlation of epigenetic ageing with telomere length. (**D**) WGCNA using mouse brain DNA methylation signatures identifies gene modules co-correlated with epilepsy and age. Rows correspond to the first principal component of a consensus co-methylation module (eigengene). Columns correspond to specific traits, including epilepsy, latency, control, and age. Each cell reports the correlation coefficient between the eigengene and trait (top) and the corresponding *adj. p-value* (bottom). Cells are colour-coded by correlation values according to the colour scale on the right; that is, strong positive correlations are denoted by strong red, and strong negative correlations by strong blue. (**E, G, I**) Module-specific heatmaps of DNA methylation profiles and unsupervised hierarchical cluster analysis. Samples representing individual animals (columns) were clustered based on their relative methylation eigengene values. Colour codes match tissue type, trait, and chronological age. Animals are consistently numbered across all heatmaps. (**F, H, J**) Functional annotations of modules based on Reactome pathways (nodes) and gene overlap between pathway annotations (edges). Node size scales with the number of genes contributing to each pathway, and edge width is proportional to the degree of gene overlap between pathways. Only parent terms are displayed for readability. ⁎ *adj. p* < 0.05. d, day; n.s., not significant.

This temporal sequence was supported by an orthogonal, non-methylation-based ageing marker. Telomere length in the mouse brain was significantly reduced in epileptic animals (n = 6, Dunn test with Benjamini-Hochberg correction, *adj. p* < 0.01, Fig. 3B). Latency animals showed a trend towards shorter telomeres, consistent with the trend in accelerated epigenetic ageing (n = 7, *p* = 0.05, Fig. 3B). Telomere length was significantly correlated with epigenetic age (n = 16, Spearman ρ = −0.56, *p* < 0.05, Fig. 3C).

In epileptic mice, brain age acceleration did not significantly correlate with the total number of seizures per animal (Spearman ρ = 0.6, *p* = 0.25; Supplementary Fig. S2D, upper panel), cumulative seizure load defined as the sum of seizure durations (ρ = 0.534, *p* = 0.30; Supplementary Fig. S2D, lower panel), or seizure frequency (ρ = 0.486, *p* = 0.36; not shown). In latency animals, brain ageing did not significantly correlate with the number of recorded spikes in the final week before termination (Spearman ρ = 0.60, *p* = 0.24; Supplementary Fig. S2E, upper panel) or across the entire recording period (ρ = 0.26, *p* = 0.66; Supplementary Fig. S2E, lower panel).

These results suggest that accelerated brain ageing also occurs in a drug-naïve experimental mTOR-driven epilepsy model, providing at least some cross-species support for the human observations.

### The converging molecular signatures of ageing and epilepsy

To investigate the molecular mechanisms underlying ageing and epilepsy, we performed weighted gene correlation network analysis (WGCNA). This approach was based on the premise that DNA methylation patterns can reflect gene regulatory networks associated with phenotypic traits (i.e., age, epilepsy, latency, and sham-control). Seven gene modules were identified from mouse brain methylation data (Fig. 3D). Two (yellow, brown) were strongly correlated with epilepsy (Pearson correlation, both *adj. p* < 0.01) and anticorrelated with the control phenotype or vice versa (*adj. p* < 0.0001 and < 0.01; Fig. 3D). Further, the brown and turquoise modules were strongly associated with age (*adj. p* < 0.0001 and < 0.01; Fig. 3D). Reactome pathway enrichment was collapsed into coherent parent functions and revealed distinct yet partially overlapping biology across significant WGCNA modules.

The yellow (*epilepsy-only*) module concentrated on growth factor-stimulated receptor-tyrosine kinase (RTK) and mitogen-activated protein kinase (MAPK) signalling, small GTPase signalling linked to cytoskeletal remodelling, axon guidance, cell–cell communication, death receptor signalling and apoptosis, as well as platelet homoeostasis, activation and signalling (Fig. 3E and F). The brown (*epilepsy & ageing*) module was likewise dominated, however less diverse regarding number of genes involved, by RTK, small GTPase, and nitric oxide signalling, cell adhesion, axon guidance and nervous system development, as well as genes involved in synaptic transmission (Fig. 3G and H). The turquoise (*ageing-only*) module extensively featured RTK/MAPK/PI3K/AKT signalling as well as representation of pathways related to small GTPase signalling and cytoskeletal remodelling, axon guidance and nervous system development, ECM organisation (e.g., integrins, collagens, proteoglycans, elastic fibre formation), mechanosensing and shear-stress response, neuroinflammation and Toll-like receptor (TLR) signalling, platelet activation and signalling, plus changes in metabolism, O-glycosylation, transmembrane transport, neuronal excitability, and ion homoeostasis (Fig. 3I and J).

In summary, all three modules converge on RTK and small GTPase signalling, axon guidance, nervous system development, and cell–cell communication. The epilepsy-only module preferentially reflects activity-dependent signalling and network remodelling, the epilepsy-and-ageing module shows a more limited overlap with additional representation of synaptic transmission, and the ageing-only module extends this shared framework to include prominent PI3K/AKT signalling, extracellular matrix remodelling, metabolic and neuroinflammatory pathways, and changes in neuronal excitability and ion homoeostasis (Fig. 3F–H, J; Supplementary Fig. S2F).

Notably, in the yellow (*epilepsy-only*) and brown (*epilepsy & ageing*) modules and respective heatmaps, mTOR-mutant nonepileptic latency mice at P42 and P80 exhibited methylation profiles resembling either sham controls or epileptic animals, suggesting a molecular signature potentially predictive of future seizure development (Fig. 3E–G). Thus, DNA methylation signatures in brain tissue may serve as early biomarkers of epileptogenesis and could possibly convey future epilepsy risk.

Taken together, brain DNA methylation signatures show promise as predictors of epilepsy risk, and converge on pathways linked to epilepsy and ageing.

## Discussion

This study identifies accelerated biological ageing as a consistent feature of the epileptic brain in structural epilepsies across human and mouse cohorts. In peripheral blood, age acceleration was more limited and heterogeneous, with a significant effect confined to FCD among the structural epilepsies, and no consistent acceleration in the genetic epilepsies examined. Brain age acceleration exceeded that of paired blood samples, except in FCD, where both tissues showed similar effects. Importantly, ageing-related signatures were already detectable before seizure onset in a somatic mTOR-driven mouse model, raising the possibility that altered epigenetic ageing contributes to epileptogenesis, although causal relationships remain to be tested.

These findings provide a molecular correlate for the increased burden of age-related disease observed in people with epilepsy, including dementia, vascular disease, metabolic and respiratory disorders, arthritis, and bone disease.^16^, ^45^, ^46^, ^47^ However, this clinical burden is likely multifactorial and may also reflect shared vascular risk factors, antiseizure medications (ASM), lifestyle, and bidirectional disease relationships, rather than a single global ageing mechanism. Epigenetic age acceleration should therefore be considered as one component of a broader vulnerability to age-related disease rather than evidence of a uniform systemic ageing process.

The marked difference between brain and blood supports a predominantly tissue-specific effect. Across structural epilepsies, age acceleration was generally greater in brain than in blood, while FCD was the only subgroup with significant age acceleration in both tissues. This provides some evidence for a systemic involvement in FCD but also suggests that the relative contribution of brain-specific and systemic ageing differs between epilepsy aetiologies. Subtle peripheral effects may nevertheless have remained undetected because of limited subgroup sizes, group imbalance, and the sensitivity of blood-based measures. Larger, longitudinal studies with matched samples across tissues will be required to determine the extent to which biological ageing in epilepsy remains brain-restricted or reflects broader systemic processes.

Because the human data were retrospective and cross-sectional, they cannot establish when ageing-related changes arise relative to epilepsy onset. We therefore used an *in utero* electroporation model of somatic mTOR activation as an aetiologically matched model of FCD type 2 rather than as a general model of epilepsy.^40^, ^48^ The model reproduces brain somatic mosaicism for a pathogenic mTOR variant, focal cytoarchitectural abnormalities, SMI32-positive dysmorphic neurons, disrupted cortical lamination, mTOR pathway activation, and a latency period before spontaneous seizures.^48^, ^49^ In this setting, age-acceleration signatures were already detectable in drug-naïve animals before seizure onset, demonstrating that neither ASM exposure nor recurrent seizures is required for their emergence. The distinct pre-seizure methylation profiles further raise the possibility that DNA methylation patterns could help stratify seizure risk before clinical onset. Relative telomere length, an independent non-methylation-based marker of biological ageing, was likewise reduced before seizure onset and correlated with methylation-based ageing.

The temporal resolution of these experiments remains limited. Two sampling time points cannot establish whether epigenetic age acceleration develops gradually, abruptly, or dynamically, nor whether early ageing-related changes are necessary or sufficient for later seizures. Nevertheless, latency animals showed heterogeneous methylation profiles resembling either sham or epileptic animals, and those with more epilepsy-like profiles also showed marked biological age acceleration. Brain age acceleration was additionally positively associated with seizure burden in epileptic animals and with spiking activity in prolonged-latency animals, although these analyses were underpowered. Together, these observations suggest that altered epigenetic ageing can precede overt epilepsy and may mark an early disease-relevant state. Whether it contributes causally to epileptogenesis will require longitudinal and perturbation studies.

Network analysis further suggested that epilepsy and ageing converge on overlapping biological processes. Epilepsy- and ageing-associated methylation modules implicated receptor tyrosine kinase and RHO-family small GTPase signalling, pathways involved in neuronal migration, axon guidance, synapse formation, and activity-dependent spine remodelling.^50^, ^51^ Epilepsy-associated modules were additionally enriched for cytoskeletal remodelling, axon guidance, cell–cell communication, and apoptosis, consistent with activity-driven network reorganisation, aberrant circuit refinement, and glial responses described in structural epilepsies.^52^ Ageing-associated modules more prominently involved PI3K/AKT signalling, extracellular matrix remodelling, immune and inflammatory processes, and altered excitability and ion homoeostasis. These findings suggest that epilepsy and ageing engage overlapping plasticity-related networks but may bias them differently, towards maladaptive rewiring in epilepsy and increasing constraints on circuit plasticity with ageing. These pathway-level interpretations remain correlative, however, because DNA methylation is an indirect measure of molecular activity and array-based profiling has limited genomic and cell-type resolution.

Several methodological considerations are important when interpreting the human findings. Epigenetic clocks differ in training data, tissue specificity, species, and platform compatibility,^1^, ^53^, ^54^, ^55^ limiting direct comparisons of age estimates between different models. We therefore used a principal-component implementation of the Horvath pan-tissue clock (PCHorvath1), designed for cross-tissue and cross-platform applications, and to reduce technical noise. Most available clocks are not optimised for paediatric samples, in which biological age progresses nonlinearly.^56^, ^57^ A key limitation is therefore the age mismatch between the surgical epilepsy and autopsy control cohorts: the control interquartile range (55–71 years) did not overlap that of any patient subgroup, requiring calibration outside the age range in which the model was estimated, by a median of 18 years in TLE and 50 years in MOGHE. Because epigenetic age estimates can regress towards the calibration age distribution, this could inflate residuals in younger samples and thus bias estimates in the direction observed here. Several observations argue against this fully explaining the findings: age acceleration was reproduced in mutant and sham mice of identical chronological age, telomere length changed concordantly as a clock-independent ageing measure, and significant acceleration was also observed in TLE, the human subgroup closest in age to the controls. Nevertheless, the magnitude of human brain age acceleration should be considered approximate until validated against control tissue spanning paediatric and young-adult ages.

Medication exposure represents an additional potential confounder. Detailed lifetime ASM histories were unavailable for this retrospective, multicentre archival cohort, whereas the autopsy and blood control cohorts were ASM-naïve. This is particularly relevant for valproate (VPA), a histone deacetylase inhibitor that increases histone acetylation and has been reported to reduce DNA methylation,^58^, ^59^ potentially affecting DNA methylation-based age estimates. However, available patient-level VPA exposure was not associated with age acceleration. Exposure was also inversely distributed relative to the observed brain effects: ganglioglioma had the lowest documented exposure (3/25) yet the largest age acceleration, whereas MOGHE had the highest exposure (38/46). The diagnosis-adjusted analysis likewise showed no association. This is broadly consistent with reports that VPA monotherapy in adults with epilepsy does not alter telomere length, an independent marker of biological ageing.^60^ Thus, although VPA may have influenced age-acceleration estimates, it is unlikely to account fully for the findings. Residual confounding by incomplete ASM histories, comorbidities, disease duration, seizure frequency and severity, cumulative seizure burden and lifestyle nevertheless remains possible.

Sex was included as a covariate in the primary human models, so mean sex differences are removed from the reported residuals by construction, but tissue availability resulted in uneven sex distributions across some diagnostic subgroups. The study was not powered to detect modest sex-specific effects or interactions between sex, age, and aetiology, which will require larger, prospectively balanced cohorts. Race and ethnicity were not recorded in the archival source data. Based on the contributing centres, the study population should therefore be considered predominantly of European descent. Ancestry is thus unlikely to explain the observed subgroup differences, although generalisability to other populations remains uncertain.

The human cohorts represent selected sources of experimentally accessible material, surgical brain tissue from patients with drug-resistant focal epilepsy and blood mainly from genetically defined developmental and epileptic encephalopathies. They are therefore not representative of epilepsy as a whole. Sample sizes were determined by tissue availability and stringent inclusion criteria rather than by a priori power calculation. Post hoc sensitivity analyses indicated that the primary brain comparisons against 20 autopsy controls could detect moderate-to-large effects (Cohen’s *d* ≈ 0.75–0.83 across FCD, MOGHE, TLE, and GG), while blood comparisons against 45 controls could detect *d* ≈ 0.57–0.81 in subgroups with n ≥ 15. Smaller subgroups were underpowered, and non-significant findings in these groups should therefore be considered inconclusive rather than evidence of no effect.

All autopsy controls originated from a single contributing centre and underwent the same centralised work-up as the surgical epilepsy samples. The analysed surgical tissue was obtained from the epileptogenic region, so the spatial extent of the ageing signal remains unknown. Its presence in histologically normal temporal neocortex from TLE cases, independently assessed by two experienced neuropathologists, suggests that it is not restricted to overtly lesional tissue. Determining whether it extends to non-epileptogenic cortex or subcortical regions will require multi-region autopsy studies or validated *in vivo* surrogates of brain methylation age.

Finally, bulk-tissue analyses cannot resolve cell-type-specific changes. This is particularly relevant across histologically distinct lesions, where differences in cellular composition may contribute to methylation signals despite reference-based deconvolution. However, single-cell studies of resected drug-resistant epilepsy tissue, including FCD, TLE, and tumours, have identified senescence-associated markers in a subpopulation of enlarged cortical pyramidal neurons, providing a plausible cellular substrate for the bulk ageing signal observed here.^61^ Cell-type-resolved and spatial analyses will be required to determine which neuronal and glial populations drive the observed changes.

The experimental findings likewise derive from a single variant mTOR-driven model and should not be generalised to mechanistically distinct epilepsies. mTOR signalling has central roles in growth, metabolism, and epigenetic regulation, and replication in non-mTOR models will therefore be important. The methylation signatures identified here are also preliminary as biomarkers and require validation in larger, longitudinal cohorts before translational application.

In summary, our data identify epigenetic age acceleration as a prominent feature of drug-resistant structural epilepsies in human brain and of somatic mTOR-driven epilepsy in mice, whereas peripheral effects are more limited and aetiology-dependent. The presence of ageing-related changes before seizure onset, together with convergence on ageing- and epilepsy-associated molecular pathways, supports further investigation of biological ageing as a potentially disease-relevant process in epileptogenesis. However, causal and therapeutic implications remain speculative. Longitudinal, cell-type-resolved, and mechanistic studies across diverse epilepsy aetiologies will be required to determine whether biological ageing contributes to epilepsy development or progression and whether it can be therapeutically modified.

## Contributors

Conceptualisation: KK.

Methodology and resources: PC, MKa, KK, MS, JH, RAJ, TH, MKu, CB, TK, HH, DD, KR, RT, AGN, AB, NV, PM, JK, AS, HP, PJ, SW, CM, VB, LH, RC, IB.

Investigation: PC, MKa, KK, MS, PM, NV, AB, JK, AS.

Visualisation: PC, MKa, MS, KK, HP.

Funding acquisition: KK, IB, VB, PJ, HP.

Project administration: KK.

Supervision: KK, JRL, PJ, HP, SW, CB.

Writing—original draft: KK, PC, MKa, HP.

Writing—review, editing, and approval: all authors.

PC, MKa, and KK have accessed and verified all data.

All authors have read and approved the final version of the manuscript for publication.

## Data sharing statement

DNA methylation data and code are available on GEO, ArrayExpress, and Zenodo under accession numbers GSE185090, GSE156374, GSE227239, GSE182991, GSE191276, E-MTAB-12728, and https://zenodo.org/records/17198342. Data that cannot be shared (e.g., potentially re-identifiable clinical information and centre-dependent consent-restricted DNA methylation/genetic data) are available only in aggregated form within the article and its Supplementary material.

## Declaration of interests

Premysl Jiruska is chair of the EpiStop patient advocacy group (unpaid). Sarah Weckhuysen received consultancy fees from UCB, Xenon Pharmaceuticals, Lundbeck, Knopp Biosciences, Encoded Therapeutics, Eisai, Angelini Pharma, Roche, CloudsOfCare, and Jazz Pharmaceuticals. Katja Kobow received travel support from EAN, ILAE, SEN, DGNN, and DGKN to attend congresses and is president-elect and board member of the DGfE (unpaid). All remaining authors declare that they have no competing interests in relation to this study.

## References

[1] Horvath S. (2013). DNA methylation age of human tissues and cell types. Genome Biol.

[2] Vetter V.M., Spieker J., Sommerer Y. (2023). DNA methylation age acceleration is associated with risk of diabetes complications. Commun Med (Lond).

[3] McCartney D.L., Stevenson A.J., Walker R.M. (2018). Investigating the relationship between DNA methylation age acceleration and risk factors for Alzheimer's disease. Alzheimers Dement (Amst).

[4] Ori A.P.S., Olde Loohuis L.M., Guintivano J. (2024). Meta-analysis of epigenetic aging in schizophrenia reveals multifaceted relationships with age, sex, illness duration, and polygenic risk. Clin Epigenet.

[5] Liao P., Ostrom Q.T., Stetson L., Barnholtz-Sloan J.S. (2018). Models of epigenetic age capture patterns of DNA methylation in glioma associated with molecular subtype, survival, and recurrence. Neuro Oncol.

[6] Shireby G.L., Davies J.P., Francis P.T. (2020). Recalibrating the epigenetic clock: implications for assessing biological age in the human cortex. Brain.

[7] Dreier J.W., Laursen T.M., Tomson T., Plana-Ripoll O., Christensen J. (2023). Cause-specific mortality and life years lost in people with epilepsy: a Danish cohort study. Brain.

[8] Kamondi A., Grigg-Damberger M., Loscher W., Tanila H., Horvath A.A. (2024). Epilepsy and epileptiform activity in late-onset Alzheimer disease: clinical and pathophysiological advances, gaps and conundrums. Nat Rev Neurol.

[9] Hoffmann L., Coras R., Kobow K. (2023). Ganglioglioma with adverse clinical outcome and atypical histopathological features were defined by alterations in PTPN11/KRAS/NF1 and other RAS-/MAP-Kinase pathway genes. Acta Neuropathol.

[10] Kobow K., Jabari S., Pieper T. (2020). Mosaic trisomy of chromosome 1q in human brain tissue associates with unilateral polymicrogyria, very early-onset focal epilepsy, and severe developmental delay. Acta Neuropathol.

[11] Miranda D.M., Rosa D.V., Costa B.S. (2020). Telomere shortening in patients with drug-resistant epilepsy. Epilepsy Res.

[12] Van Loo K.M.J., Carvill G.L., Becker A.J. (2022). Epigenetic genes and epilepsy - emerging mechanisms and clinical applications. Nat Rev Neurol.

[13] Jabari S., Kobow K., Pieper T. (2022). DNA methylation-based classification of malformations of cortical development in the human brain. Acta Neuropathol.

[14] Rho J.M., Boison D. (2022). The metabolic basis of epilepsy. Nat Rev Neurol.

[15] Ribierre T., Bacq A., Donneger F. (2024). Targeting pathological cells with senolytic drugs reduces seizures in neurodevelopmental mTOR-related epilepsy. Nat Neurosci.

[16] Josephson C.B., Gonzalez-Izquierdo A., Denaxas S., Sajobi T.T., Klein K.M., Wiebe S. (2023). Independent associations of incident epilepsy and enzyme-inducing and non-enzyme-inducing antiseizure medications with the development of osteoporosis. JAMA Neurol.

[17] Vossel K.A., Tartaglia M.C., Nygaard H.B., Zeman A.Z., Miller B.L. (2017). Epileptic activity in Alzheimer's disease: causes and clinical relevance. Lancet Neurol.

[18] Galovic M., van Dooren V.Q.H., Postma T.S. (2019). Progressive cortical thinning in patients With focal epilepsy. JAMA Neurol.

[19] Pardoe H.R., Devinsky O., Robson J. (2019). Global Socioeconomic Context and Brain Ageing in Epilepsy: an ENIGMA-Epilepsy study. JAMA Neurol.

[20] Hwang G., Hermann B., Nair V.A. (2020). Brain aging in temporal lobe epilepsy: chronological, structural, and functional. Neuroimage Clin.

[21] Lehner A., Hoffmann L., Rampp S. (2024). Age-dependent increase of perineuronal nets in the human hippocampus and precocious aging in epilepsy. Epilepsia Open.

[22] Kobow K., Ziemann M., Kaipananickal H. (2019). Genomic DNA methylation distinguishes subtypes of human focal cortical dysplasia. Epilepsia.

[23] Wang D.D., Katoch M., Jabari S. (2023). The specific DNA methylation landscape in focal cortical dysplasia ILAE type 3D. Acta Neuropathol Commun.

[24] Miller-Delaney S.F., Bryan K., Das S. (2015). Differential DNA methylation profiles of coding and non-coding genes define hippocampal sclerosis in human temporal lobe epilepsy. Brain.

[25] Debski K.J., Pitkanen A., Puhakka N. (2016). Etiology matters - genomic DNA methylation patterns in three rat models of acquired epilepsy. Sci Rep.

[26] Kobow K., Kaspi A., Harikrishnan K.N. (2013). Deep sequencing reveals increased DNA methylation in chronic rat epilepsy. Acta Neuropathol.

[27] Kobow K., Khan N., Noebels J.L., Avoli M., Rogawski M.A., Vezzani A., Delgado-Escueta A.V. (2024). Jasper's basic mechanisms of the epilepsies.

[28] GBD 2016 Epilepsy Collaborators (2019). Global, regional, and national burden of epilepsy, 1990-2016: a systematic analysis for the Global Burden of Disease Study 2016. Lancet Neurol.

[29] Scheffer I.E., Berkovic S., Capovilla G. (2017). ILAE classification of the epilepsies: position paper of the ILAE Commission for Classification and Terminology. Epilepsia.

[30] Perucca E., Brodie M.J., Kwan P., Tomson T. (2020). 30 years of second-generation antiseizure medications: impact and future perspectives. Lancet Neurol.

[31] Redman L.M., Smith S.R., Burton J.H., Martin C.K., Il'yasova D., Ravussin E. (2018). Metabolic slowing and reduced oxidative damage with sustained caloric restriction support the rate of living and oxidative damage theories of aging. Cell Metab.

[32] Janssens G.E., Molenaars M., Herzog K. (2024). A conserved complex lipid signature marks human muscle aging and responds to short-term exercise. Nat Aging.

[33] Du N., Yang R., Jiang S. (2024). Anti-aging drugs and the related signal pathways. Biomedicines.

[34] Yuskaitis C.J., Modasia J.B., Schrotter S. (2022). DEPDC5-dependent mTORC1 signaling mechanisms are critical for the anti-seizure effects of acute fasting. Cell Rep.

[35] Wong M. (2011). Rapamycin for treatment of epilepsy: antiseizure, antiepileptogenic, both, or neither?. Epilepsy Curr.

[36] Barba C., Blumcke I., Winawer M.R. (2023). Clinical features, neuropathology, and surgical outcome in patients With refractory epilepsy and brain somatic variants in the SLC35A2 gene. Neurology.

[37] Blumcke I., Aronica E., Miyata H. (2016). International recommendation for a comprehensive neuropathologic workup of epilepsy surgery brain tissue: a consensus Task Force report from the ILAE Commission on Diagnostic Methods. Epilepsia.

[38] Wang Y., Yu T., Blumcke I. (2023). The clinico-pathological characterisation of focal cortical dysplasia type IIb genetically defined by MTOR mosaicism. Neuropathol Appl Neurobiol.

[39] Honke J., Hoffmann L., Coras R. (2023). Deep histopathology genotype-phenotype analysis of focal cortical dysplasia type II differentiates between the GATOR1-altered autophagocytic subtype IIa and MTOR-altered migration deficient subtype IIb. Acta Neuropathol Commun.

[40] Najm I., Lal D., Alonso Vanegas M. (2022). The ILAE consensus classification of focal cortical dysplasia: an update proposed by an ad hoc task force of the ILAE diagnostic methods commission. Epilepsia.

[41] Blumcke I., Thom M., Aronica E. (2013). International consensus classification of hippocampal sclerosis in temporal lobe epilepsy: a Task Force report from the ILAE Commission on Diagnostic Methods. Epilepsia.

[42] Murthy M., Shireby G., Miki Y. (2023). Epigenetic age acceleration is associated with oligodendrocyte proportions in MSA and control brain tissue. Neuropathol Appl Neurobiol.

[43] Zhou W., Hinoue T., Barnes B. (2022). DNA methylation dynamics and dysregulation delineated by high-throughput profiling in the mouse. Cell Genom.

[44] Charan J., Kantharia N.D. (2013). How to calculate sample size in animal studies?. J Pharmacol Pharmacother.

[45] Giussani G., Bianchi E., Beretta S. (2021). Comorbidities in patients with epilepsy: Frequency, mechanisms and effects on long-term outcome. Epilepsia.

[46] St Germaine-Smith C., Liu M., Quan H., Wiebe S., Jette N. (2011). Development of an epilepsy-specific risk adjustment comorbidity index. Epilepsia.

[47] Wiebe S., Hesdorffer D.C. (2007). Epilepsy: being ill in more ways than one. Epilepsy Curr.

[48] Kim J.K., Cho J., Kim S.H. (2019). Brain somatic mutations in MTOR reveal translational dysregulations underlying intractable focal epilepsy. J Clin Invest.

[49] Lim J.S., Kim W.I., Kang H.C. (2015). Brain somatic mutations in MTOR cause focal cortical dysplasia type II leading to intractable epilepsy. Nat Med.

[50] Wu X., Yang Z., Zou J. (2025). Protein kinases in neurodegenerative diseases: current understandings and implications for drug discovery. Signal Transduct Target Ther.

[51] Govek E.E., Newey S.E., Van Aelst L. (2005). The role of the Rho GTPases in neuronal development. Genes Dev.

[52] Francois L., Romagnolo A., Luinenburg M.J. (2024). Identification of gene regulatory networks affected across drug-resistant epilepsies. Nat Commun.

[53] Zheng Z., Li J., Liu T. (2024). DNA methylation clocks for estimating biological age in Chinese cohorts. Protein Cell.

[54] Lu A.T., Fei Z., Haghani A. (2023). Universal DNA methylation age across mammalian tissues. Nat Aging.

[55] Hannum G., Guinney J., Zhao L. (2013). Genome-wide methylation profiles reveal quantitative views of human aging rates. Mol Cell.

[56] Musci R.J., Raghunathan R.S., Johnson S.B. (2023). Using epigenetic clocks to characterize biological aging in studies of children and childhood exposures: a systematic review. Prev Sci.

[57] Higgins-Chen A.T., Thrush K.L., Wang Y. (2022). A computational solution for bolstering reliability of epigenetic clocks: implications for clinical trials and longitudinal tracking. Nat Aging.

[58] Milutinovic S., D'Alessio A.C., Detich N., Szyf M. (2007). Valproate induces widespread epigenetic reprogramming which involves demethylation of specific genes. Carcinogenesis.

[59] Tremolizzo L., Difrancesco J.C., Rodriguez-Menendez V. (2012). Valproate induces epigenetic modifications in lymphomonocytes from epileptic patients. Prog Neuropsychopharmacol Biol Psychiatry.

[60] Sanchez-Badajos S., Ortega-Vazquez A., Lopez-Lopez M., Monroy-Jaramillo N. (2025). Valproic acid and lamotrigine differentially modulate the telomere length in epilepsy patients. J Clin Med.

[61] Ge Q., Yang J., Huang F. (2025). Multimodal single-cell analyses reveal molecular markers of neuronal senescence in human drug-resistant epilepsy. J Clin Invest.

