# Supplementary material for: Epigenetic age acceleration in the brain and blood of people with epilepsy and in a mouse model of focal cortical dysplasia: a retrospective cross-sectional study
Source: eBioMedicine. 2026 Nov 9;134:106550. doi: 10.1016/j.ebiom.2026.106550 (PMC13770574; doi:10.1016/j.ebiom.2026.106550)
Supplement: Supplementary file 1 [file mmc1.docx]

**Supplementary Materials**

Content

1. Method extension
2. Supplementary References
3. Supplementary Figures 1 and 2 and Figure Legends

**1. Method extension**

***In-utero* electroporation and FCD induction**

The FCD lesion was induced by *in-utero* electroporation ^1-3^. Time-pregnant mice (E14.5) were anesthetized with Isoflurane in oxygen (4% Isoflurane for induction, 2% for maintenance). The abdomen was shaved and wiped with 70% ethanol. A midline laparotomy was performed, and uterine horns were exposed on sterile gauze. Except for the embryos near the cervix, each embryo was injected into the left lateral ventricle with a mixture of plasmids diluted in 2 μg/ml Fast Green (F7252, Sigma, USA) in dH_2_O. The injection was performed using pulled glass capillaries ground into a beveled angle (the outer diameter of the shaft close to the opening was 50-60 µm). Sham control animals were electroporated with the plasmid containing CAG-iRFP713 (1,5 μg/μl) only, whereas FCD animals received a mixture of plasmids containing pCAG-mTORpL2427P-IRES-mAmetrine (3 μg/μl; SoVarGen Co., Ltd., South Korea), and CAG-iRFP713 (1,5 μg/μl). Sham-treated therefore controlled for surgical, electroporation, and implantation effects in the absence of the FCD-inducing mutation. Just after the injection, electroporation of plasmids was performed using a custom-made electroporator with a 40 mA current limiter, delivering five square pulses of 35-45 V/50 ms with 950 ms inter-stimulus intervals. Pulses were delivered by forceps-type electrodes (3 mm, CUY650P3, Nepagene) positioned on each embryo's head (+ pole placed above the left somatosensory cortex). After electroporation, the uterus was reinserted into the abdominal cavity, the muscles and skin were sutured, and the wound was treated with a disinfectant liquid plaster. Ketoprofen (5 mg/kg, s.c.) was applied for three consecutive days, including the day of the surgery. At P0-1, the pups were screened for the presence of the lesion by examining reporter protein fluorescence through the skin. Pups with verified fluorescent lesions were left with their mothers until weaning.

**Electrode implantation and EEG recording**

Mice were implanted with EEG electrodes at P35 ± 1, followed by a 5-day recovery period. Before implantation, custom EEG implants were prepared by soldering coated silver wires (127 μm in diameter, AM Systems, Inc., USA, cat. no. 786000) to prefabricated connectors (TME Electronic Components, Poland, cat. no. DS1065-03-2*6S8BV). Mice were anaesthetized with Isoflurane (4% for induction, 2% for maintenance), and were administered buprenorphine (0.1 mg/kg, s.c.). A small area of skin on the top of the head was removed to expose the frontal and parietal bones of the skull, and fluorescence screening was performed to determine the exact position of the lesion. Six holes, adjusted to the position of the lesion, were drilled through the skull. One electrode was placed in the centre of the lesion ("L"). One electrode was implanted contralaterally, homotopic to the lesion electrode (contralateral, "C"), and two electrodes over the frontal cortices (frontal left, "FL", and frontal right, "FR"). Two holes were drilled above the cerebellum for grounding/reference electrodes. The electrodes were positioned on the surface of the dura mater, and the holes were covered with bone wax (SMI, Belgium, cat. no. Z046). The electrodes and connector were attached to the skull using cyanoacrylate glue (Loctite, USA, cat. no. 1363589). During a five-day recovery period mice were receiving ketoprofen (5 mg/kg, s.c.). Animals were then individually connected to video-EEG for continuous monitoring for at least 2 days for animals in the P42 latency period group and at least 20 days (on average 36 days) for animals in the P80 groups. Recordings were performed through wires plugged into the head-mount, which was connected to the recording device via counterbalanced slip rings. The spontaneous electrographic activity was amplified, band-pass filtered (0.1 Hz-1.6 kHz), and sampled at 5 kHz using a 32-channel head stage amplifier with AD converter (Intan Technologies, USA, cat. no. RHD2132) and Spike2 software (Cambridge Electronic Design, Cambridge, UK). The EEG signal was analyzed using Spike2 and custom-made scripts in a Matlab computing environment (Mathworks Inc., USA). Seizures were confirmed by visual review of the EEG recordings and classified as such when rhythmic epileptiform discharges lasted >10 s and were accompanied by a corresponding behavioral correlate on video. Interictal epileptiform discharges (IEDs) were quantified in latency animals (P80) in the EEG channel located within the lesion using a Hilbert envelope-based detector with the original settings described previously ^4^. IED rates were calculated as the number of detected events divided by the total duration of available EEG recordings for the respective analysis period (last 7 days versus entire recording), thereby accounting for occasional recording dropouts.

**Mouse tissue preparation**

Each mouse was deeply anaesthetized with ketamin+xylazine at a dose of (150 mg/kg ketamine and 12.5 mg/kg xylazine, s.c.). The absence of the pedal withdrawal reflex confirmed anaesthesia depth. The blood collection was performed carefully to avoid hemolysis and preliminary clotting. The thoracic cavity was opened, and the heart was exposed. A 25-gauge needle attached to a 2 mL syringe was used to puncture the right ventricle, and blood was drawn slowly to avoid collapsing the heart. Up to 1.5 mL of blood was collected per mouse and immediately stored at -80 °C. Genomic DNA was automatically extracted from 200 µl whole blood using the EZ1&2 DNA Blood Kit (Qiagen, Germany) and EZ1 extraction robot according to the manufacturer's instructions. Mice were decapitated after blood collection, and the whole brain was promptly dissected out and sliced into 400 μm-thick sections in cooled (4 °C) oxygenated PBS. Slices were collected so that they spanned the cortical lesion. The first and the third slices were embedded in O.C.T (VWR Q Path O.C.T. Compound Mounting Medium), frozen instantly on dry ice, and stored at -80 °C. The second and the fourth slices were fixed in 3 % PFA at room temperature for a minimum of 12 h to prepare formalin-fixed paraffin-embedded (FFPE) samples. DNA was extracted from a cryopreserved coronary hemispheric brain slice. Mouse blood samples were processed as described before for human blood.

**2. Supplementary References**

1. Lim JS, Kim WI, Kang HC, et al. Brain somatic mutations in MTOR cause focal cortical dysplasia type II leading to intractable epilepsy. *Nat Med* 2015;21:395-400.

2. Prochazkova N, Nguyenova MT, Rehorova M, et al. NeuroPorator: An open-source, current-limited electroporator for safe in utero gene transfer. *J Neurosci Methods* 2024;406:110126.

3. Procházková N, Olson CVL, Shariati N, et al. Seizure occurrence in FCD type II is predicted by lesion position and linked to cytoarchitectural alterations. *Acta Neuropathol Commun* 2025;13:251.

4. Janca R, Jezdik P, Ebel M, et al. Distinct patterns of interictal intracranial EEG in focal cortical dysplasia type I and II. *Clin Neurophysiol* 2023;151:10-17.

**3. Supplementary Figures**

**Supplementary Figure 1**

**Legend to Supplementary Fig. 1:** (**A**) Representative histology of epilepsy patients with structural brain lesions included in this study. CD34 - progenitor cell antigen (diagnostic biomarker for ganglioglioma), H&E - hematoxilin and eosin, HC - hippocampus, FCD - focal cortical dysplasia, GG - ganglioglioma, MAP2 - marker of mature neurons, stains neuronal dendrites and perikarya (staining indicates blurred grey-white matter junction due to heterotopic neurons in the deep white matter), MOGHE - mild malformation of cortical development with oligodendroglial hyperplasia in epilepsy, NCx - neocortex, NeuN - marker of mature neurons, stains neuronal cell bodies and nuclei (staining indicates severe segmental neuronal cell loss in the hippocampus, asterisks), OLIG2 - mature oligodendrocyte marker (staining indicates increased oligodendroglial cell density), SMI32 - marker of non-phosphorylated neurofilament H (staining highlights presence of dysmorphic neurons), TLE - temporal lobe epilepsy with hippocampal sclerosis, VIM - marker of balloon cells (arrows). (**B-D**) Benchmarking of 4 clocks in human brain (i.e., no-epilepsy autopsy controls) and blood control samples (healthy no-epilepsy individuals). RRSE - Robust Residual Standard Error; RMSE - Root Mean Square Error; y - year

**Supplementary Figure 2**

**Legend to Supplementary Fig. 2:** (**A**) Study design summarizing model induction, electrode implantation, seizure monitoring, and tissue collection at P42 and P80. A unilateral FCD type 2 lesion was induced by in utero co-electroporation of plasmids carrying mTOR mutation and iRFP. (**B**) Whole brain section of an mTOR mutant mouse with FCD lesion. Immunofluorescent (upper panel) and DAB-labeling (lower panel) of SMI32 highlighting dysmorphic neurons in the center of the lesion (red circled). (**C**) iRFP fluorescence visualizes the extent of the FCD lesion. Four electrodes were implanted in each animal (FR - frontal right, FL - front left, L - lesion, C - contralateral). Black dots mark the electrode positions. A representative seizure trace example is shown. (**D**) Epigenetic age acceleration in epilepsy animals in dependence of the number of seizures (upper panel) and cumulative seizure load (i.e., sum of seizure durations; lower panel). (**E**) Epigenetic age acceleration in latency animals in dependence of the number of lesional IEDs in the final week before termination (upper panel) and over the entire recording period (lower panel). (**F**) Yellow, brown, and turquoise module-specific pathway enrichment identified with WGCNA from brain DNA methylation patterns in the mTOR mouse model. Circos plot showing pathway enrichment and functional overlap across modules at different cut-offs (adj*. p* < 0.01). E - embryonic day; d - day; h - hour; IED - interictal epileptiform discharges; IUE - in utero electroporation; mTOR - mammalian target of rapamycin; P - post-natal day; s - seconds; sz - seizures.
